# Supplementary material for: Inducible caspase 9-mediated suicide gene therapy using AAV6 vectors in a murine model of breast cancer
Source: Mol Ther Methods Clin Dev. 2023 Nov 24;31:101166. doi: 10.1016/j.omtm.2023.101166 (PMC10750187; doi:10.1016/j.omtm.2023.101166)
Supplement: Document S1. Figures S1–S4 [file mmc1.pdf]

OMTM, Volume *31*

Supplemental information

Inducible caspase 9-mediated suicide gene therapy using AAV6 vectors in a murine model of breast cancer

Subhajit Pathak, Vijayata Singh, Narendra Kumar, and Giridhara R. Jayandharan

SUPPLEMENTAL DATA

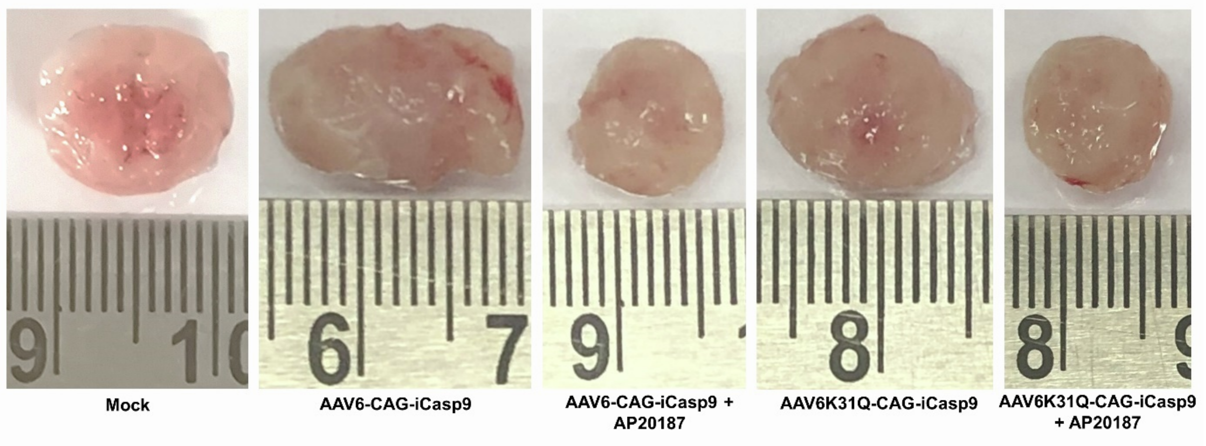

Figure S1. Morphology of breast cancer tumors after suicide gene therapy with AAV6-iCasp9 vectors. Allografted mice with an average tumor volume of ~100-150 mm^3 were administered with AAV6-iCasp9 vectors (5×10^{10} vgs/animal) with or without AP20187 and PBS (mock-treated group). Representative tumor tissues harvested from animals ten days after suicide gene transfer are shown.

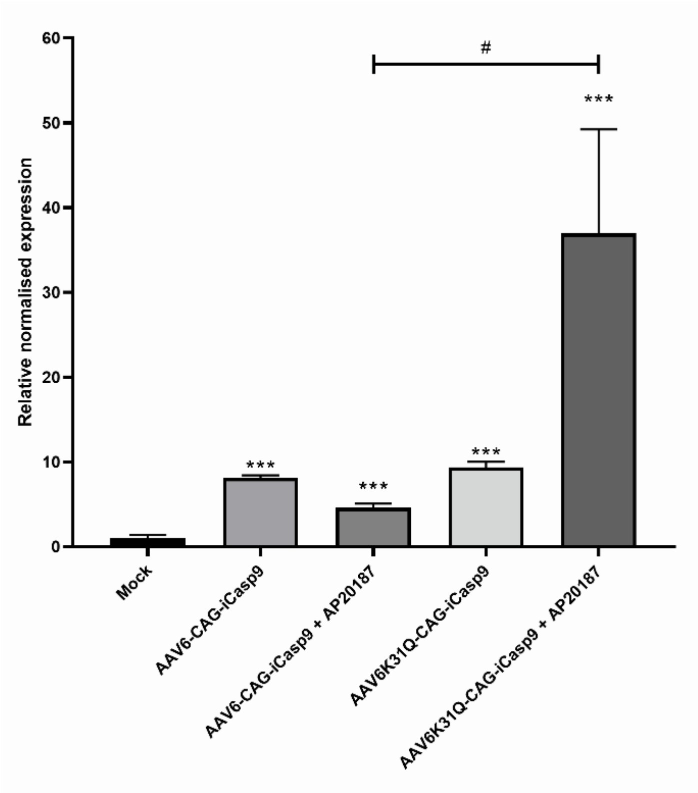

Figure S2. Levels of *iCasp9* after AAV vector administration in breast cancer tissue. An increase in *iCasp9* mRNA levels was observed in breast tumor tissues after administration with AAV6-iCasp9 vectors. Data are from 3-6 technical replicates per group and represented as mean ± SEM. *** $p \leq 0.001$ *vs* mock; # $p \leq 0.05$ *vs* AAV6-CAG-iCasp9 + AP20187.

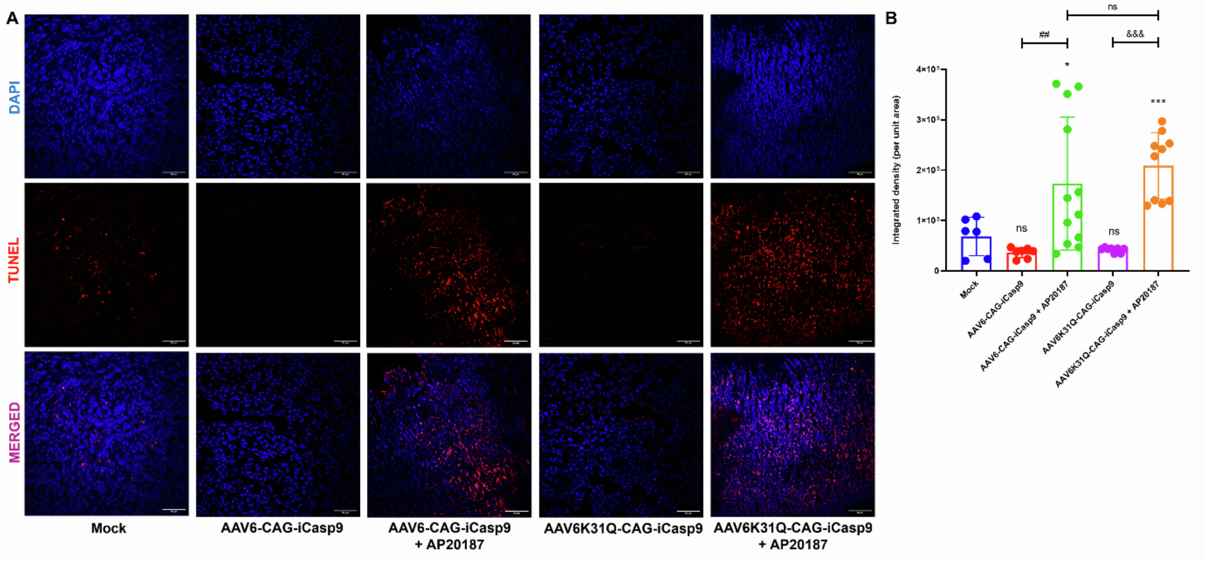

Figure S3. Detection of apoptosis by DNA damage in breast cancer tissue. Ten days after receiving the gene therapy, female athymic$^{nu/nu}$ mice with 4T1 tumors were euthanized. TUNEL assay was performed after surgical removal, fixation, and sectioning of tumors. (A) Micrographs representing the treatment groups (AAV6-CAG-iCasp9 and AAV6K31Q-CAG-iCasp9) with AP20187 showed a clear distribution of apoptotic cells within the tumors when compared to mock-treated animals (magnification 40X, scale bar 50 µm). In AAV6-CAG-iCasp9 and AAV6K31Q-CAG-iCasp9 vector-only administered mice, the absence of AP20187 failed to induce apoptosis. (B) The number of TUNEL-positive cells between various treatment groups was quantified using ImageJ software (* $p \leq 0.05$, *** $p \leq 0.001$, not significant (ns) *vs* mock group; ## $p \leq 0.01$ *vs* AAV6-CAG-iCasp9; &&& $p \leq 0.001$ *vs* AAV6K31Q-CAG-iCasp9). Data are represented as mean ± SD. The data are from 6-12 sections from each treatment group.

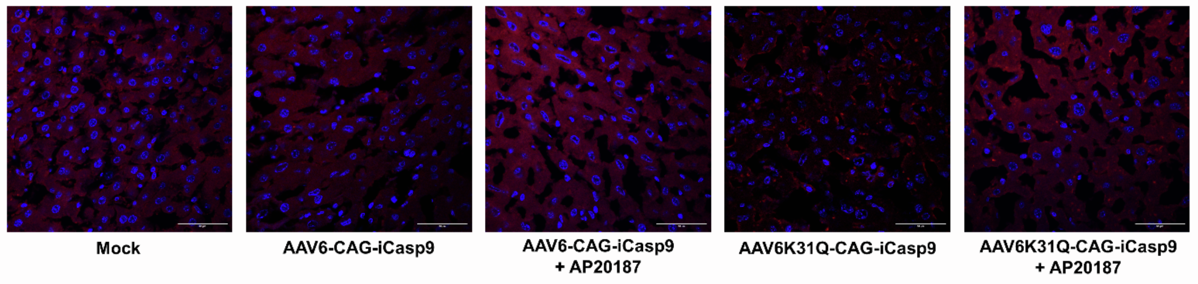

Figure S4. Evaluation of off-target Caspase 9 protein expression in the liver tissue. Liver tissues were harvested from all the treatment groups, and immunohistochemistry was performed as discussed in methods section. The micrographs of the liver sections did not show the activation of Caspase 9 protein in any of the treatment groups (magnification 63X, scale bar 50 µm).
