# Supplementary material for: Inducible caspase 9-mediated suicide gene therapy using AAV6 vectors in a murine model of breast cancer
Source: Mol Ther Methods Clin Dev. 2023 Nov 24;31:101166. doi: 10.1016/j.omtm.2023.101166 (PMC10750187; doi:10.1016/j.omtm.2023.101166)
Supplement: Document S2. Article plus supplemental information [file mmc2.pdf]

Molecular Therapy
Methods & Clinical Development
Original Article

Inducible caspase 9-mediated suicide gene therapy using AAV6 vectors in a murine model of breast cancer

Subhajit Pathak,[1] Vijayata Singh,[1] Narendra Kumar,[1] and Giridhara R. Jayandharan[1]

[1]Laurus Center for Gene Therapy, Department of Biological Sciences and Bioengineering and Mehta Family Center for Engineering in Medicine and Gangwal School of Medical Sciences and Technology, Indian Institute of Technology, Kanpur, Uttar Pradesh 208016, India

Breast carcinoma has one of the highest incidence rates (11.7%), with significant clinical heterogeneity. Although conventional chemotherapy and surgical resection are the current standard of care, the resistance and recurrence, after these interventions, necessitate alternate therapeutic approaches. Cancer gene therapy for breast cancer with the suicide gene is an attractive option due to their directed delivery into the tumor. In this study, we have developed a novel treatment strategy against breast cancer with recombinant adeno-associated virus (AAV) serotype 6 vectors carrying a suicide gene, inducible *Caspase 9* (*iCasp9*). Upon treatment with AAV6-iCasp9 vectors and the chemical inducer of dimerizer, AP20187, the viability of murine breast cancer cells (4T1) was significantly reduced to ∼40%–60% (mock control 100%). Following intratumoral delivery of AAV6-iCasp9 vectors in an orthotopic breast cancer mouse model, we observed a significant increase in *iCasp9* transgene expression and a significant reduction in tumor growth rate. At the molecular level, immunohistochemical analysis demonstrated subsequent activation of the effector caspase 3 and cellular death. These data highlight the potential of AAV6-iCasp9-based suicide gene therapy for aggressive breast cancer in patients.

INTRODUCTION

The most reported malignancy in females that results in significant mortality is breast carcinoma.[1] In 2020, the incidence of breast cancer among all populations is ∼2.2 million, positioning it first among all cancer types.[2] The global burden of breast cancer is likely to reach ∼3.19 million by 2040.[2] Clinically, breast cancer is heterogeneous and divided into three major subtypes: estrogen receptor (ER)–positive and progesterone receptor (PR)–positive, human epidermal growth factor receptor-2$^+$ and triple-negative breast cancer (TNBC).[3] Drug resistance is a crucial contributing factor mainly associated with the recurrence and relapse of breast cancer.[4] Therefore, there is a high demand for supplementing the currently available therapy for breast cancer. In addition to immunotherapy, an effective therapeutic approach is gene therapy, which has shown promise in breast cancer patients at all stages.[4,5]

Cancer gene therapy involves delivering the potential therapeutic transgene to the cancer cells to trigger apoptosis and a bystander effect in the heterogeneous tumor tissue.[6] In addition to several gene therapy–based techniques, such as oncogene inhibition, tumor suppressor gene activation, and antiangiogenic therapy, one of the effective methods is suicide gene therapy.[7,8] Broadly, suicide gene therapy is categorized into enzyme-activating prodrug therapy and gene-inducing cytotoxicity upon drug treatment.[7] Herpes simplex virus thymidine kinase (HSV-TK) and cytosine deaminase are the two transgenes in enzyme-activated prodrug treatment that have been the most explored.[9–11] However, certain limitations, such as the immunogenic nature of the viral and bacterial transgenes, along with the dependency of the activated drug on the cell cycle, can affect the phenotypic rescue.[12] An alternative strategy of using an inducible *Caspase 9* (*iCasp9*) transgene, a cytotoxic synthetic analog to mammalian *Casp9* gene, linked with an FK506 binding protein (FKBP) of human origin, has been used to silence genes during T cell therapy.[13] Chemical inducer of dimerizer, AP20187, a biologically nonreactive small molecule, causes the dimerization of iCasp9 upon binding to the FKBP domain.[14] In addition, the cytotoxic effect of the iCasp9/AP20187 combination is not reliant on the cell-cycle phase, which can be useful in a heterogeneous tissue such as breast cancer tissue. A recent study has demonstrated the effectiveness of the iCasp9/AP20187 combination in inducing cytotoxicity in several breast cancer cell lines.[15] Adeno-associated virus (AAV) is a highly effective gene delivery vector for treating various monogenic and complex disorders.[16] It offers excellent potential in current therapeutic-gene-based delivery in cancer studies because it is nonpathogenic and has low immunogenicity.[17,18] We have previously demonstrated the utility of AAV2 and AAV6-based *iCasp9* vector systems in hepatocellular carcinoma (HCC) and acute myeloid leukemia models.[19–21] Previous reports have highlighted the role of posttranslational modifications on AAV capsid and their impact on vector transduction.[22] Neddylation is a ubiquitin-like modifier of the target proteins.[23] A previous study has shown the involvement of Neddylation in the

Received 21 July 2023; accepted 20 November 2023;
https://doi.org/10.1016/j.omtm.2023.101166.

Correspondence: Giridhara R. Jayandharan, Laurus Center for Gene Therapy, Department of Biological Sciences and Bioengineering & Mehta Family Center for Engineering in Medicine and Gangwal School of Medical Sciences and Technology, Indian Institute of Technology, Kanpur, Uttar Pradesh 208016, India.
E-mail: jayrao@iitk.ac.in

This is an open access article under the CC BY license (http://creativecommons.org/licenses/by/4.0/).

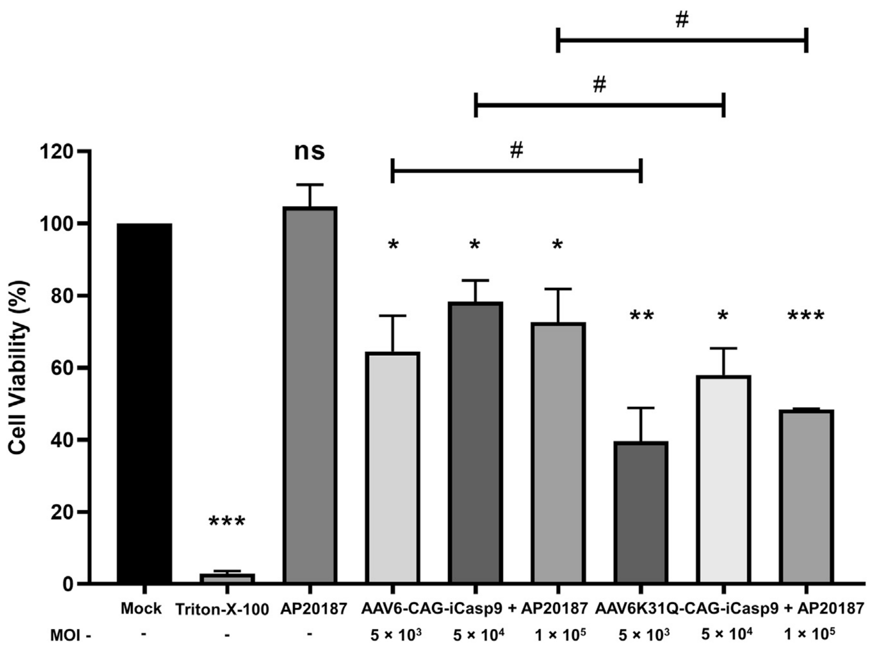

Figure 1. Cytotoxic efficiency of AAV6-iCasp9 vectors *in vitro*

AAV6 vectors encoding the *iCasp9* gene were used to infect breast cancer cells (4T1) at MOIs of 5×10^3, 5×10^4, and 1×10^5 (3 replicates each). The cytotoxicity of the vector-transduced cells was subsequently measured by MTT assay. Triton X-100 was used as a positive control. Data are represented as mean ± SD. *p $\leq$ 0.05; **p $\leq$ 0.01; ***p $\leq$ 0.001; ns (not significant) versus mock; and $^{\#}$p $\leq$ 0.05 versus AAV6-CAG-iCasp9 +AP20187. No significant difference in cell viability was observed when cells were treated with increasing MOI of the same vector.

modulation of the HSV-1 life cycle.[24] We have predicted several Neddylation posttranslational modification sites in the capsid protein of AAV2 vectors. Further abolition of these sites resulted in an improved therapeutic outcome during retinal and hepatic gene therapy.[25,26] In the present study, we used an AAV6 vector for suicide gene therapy because it has shown significant potential in mitigating the neu-positive murine breast cancer *in vivo*.[27] It must be noted that capsid-modified AAV6 vectors carrying reporter transgenes have been previously reported to have higher transduction in a luminal A type (ER^+/PR^+) T47D breast cancer cell line.[28] The present study involves the testing and characterization of both the AAV6 wild type (WT) and an AAV6 K31Q Neddylation mutant vector based on the *iCasp9* system for suicide gene therapy in a murine model of breast cancer.

RESULTS

Recombinant AAV vector induces cytotoxicity in breast cancer cells

We first packaged the *iCasp9* transgene under the regulation of strong ubiquitous promoter cytomegalovirus (CMV) early enhancer/chicken-β-actin (CAG) promoter into AAV serotype 6 WT (AAV6-CAG-iCasp9) and AAV6 K31Q Neddylation mutant (AAV6K31Q-CAG-iCasp9) vectors to evaluate their cytotoxic potential. A qPCR approach was used to estimate the physical particle titers of the vectors (vector genomes [vg]/mL).[29] To ascertain cytotoxicity, a highly invasive and tumorigenic mouse mammary carcinoma cell line, 4T1, was chosen.[30] The 4T1 cells were infected at a range of MOI of 5×10^3, 5×10^4, and 1×10^5 with AAV6-CAG-iCasp9 or AAV6K31Q-CAG-iCasp9 vectors. Next day, the vector-infected 4T1 cells were treated with 10 nM of AP20187. Cell survival was determined 24 h later using an absorbance-based MTT assay kit. Our data (Figure 1) revealed that the cell survivability in AAV6-CAG-iCasp9-infected cells was ~64%–78% at different MOI, whereas AAV6K31Q-CAG-iCasp9-treated cells had ~40%–58% viability. It is evident that in all AAV6-iCasp9 vector-treated groups, cell viability was significantly (AAV6-CAG-iCasp9: p $\leq$ 0.05 [all MOIs]; AAV6K31Q-CAG-iCasp9: p $\leq$ 0.05 [MOI 5×10^3], p $\leq$ 0.01 [MOI 5×10^4], p $\leq$ 0.001 [MOI 1×10^5]) decreased compared to mock-treated cells (cell viability 100%). Furthermore, we also observed that the AAV6K31Q Neddylation mutant vector–treated group had very high cell kill (~20%, p $\leq$ 0.05) when compared to the AAV6 WT vector at different MOI. These results suggested that the AAV6-iCasp9 vectors are able to induce cytotoxicity in 4T1 cells and that the AAV6 K31Q Neddylation mutant vector has higher transduction efficiency *in vitro* when compared to the AAV6 WT vector, as observed in other AAV Neddylation mutant vectors in our previous studies.[25,26] However, it was observed that cell cytotoxicity did not exhibit a proportional increase with increasing vector MOI, which could be a result of a potential saturation dose effect. Once the virus successfully infiltrates the host cell, the subsequent cell death, due to the expression of the suicide gene, becomes independent of the quantity of entering viruses. This implies that only a minimum threshold level of vector presence is required within the cell to trigger the cytotoxic response; any increase in MOI beyond this critical point does not alter the phenotype (cell death). Similar observations were made in a study by Li et al., in which almost no difference was found between MCF-7 cell survival as AAV-2/TRE/HSVtk/Tet-On infection reached a plateau when rAAV titers ranged from 10^4 to 10^6 vector particles/cell.[10]

AAV6 vector-mediated suicide gene therapy reduces tumor growth in murine model of breast cancer

We established an orthotopic model of breast cancer in female athymic$^{nu/nu}$ mice. Subsequently, the cytotoxic effect of AAV6-CAG-iCasp9 and AAV6K31Q-CAG-iCasp9 vectors was assessed in these murine models *in vivo*. After administration of ~1×10^6 4T1 cells, the transplanted animals developed visible tumor nodules within the mammary fat pad starting from day 4. After 7 days, when the tumors reached 100–150 mm^3 in volume, ~5×10^{10} vg of AAV6-iCasp9 vectors or PBS in a 100-μL volume was injected intratumorally

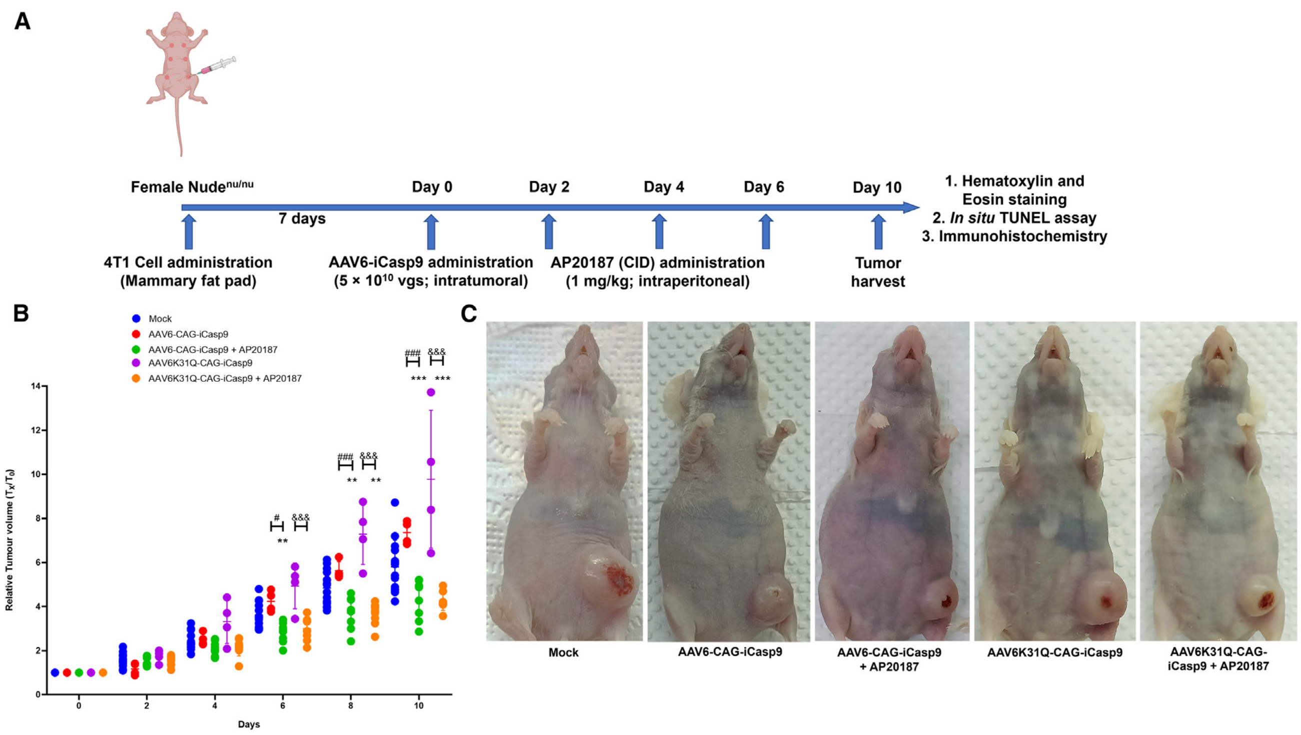

Figure 2. Antitumorigenic effect of AAV6-iCasp9 vectors (WT and K31Q mutant) with AP20187 dimerizer drug in an orthotopic model of breast cancer

(A) An orthotopic allograft breast cancer model was developed by administering the 4T1 cell line into the mammary fat pad of female athymic$^{nu/nu}$ mice. When the tumor size reached a volume of ~100–150 mm^3, AAV vectors were administered intratumorally. On days 2, 4, and 6 post-vector administration, AP20187 was administered intraperitoneally. Tumors were harvested at day 10 post-vector administration for further molecular and biochemical assays. $^{**}p \leq 0.01$; $^{***}p \leq 0.001$ versus mock group; $^{\#}p \leq 0.05$, $^{\#\#\#}p \leq 0.001$ versus AAV6-CAG-iCasp9; $^{\&\&\&}p \leq 0.001$ versus AAV6K31Q-CAG-iCasp9. (B) Animals from the AAV6-CAG-iCasp9 and AAV6K31Q-CAG-iCasp9 groups with AP20187 treatment had a more attenuated tumor growth rate than mock-injected and AAV6-iCasp9 vector-only animals ($p \leq 0.001$). Data are represented as mean ± SD. (C) Representative animals at day 10 from each experimental group are depicted.

(Figure 2A). Intraperitoneal injection of AP20187 (1 mg/kg, three doses) was given to each mouse at 48-h intervals starting the next day.[31] Mice injected with the AAV6-CAG-iCasp9 and AAV6K31Q-CAG-iCasp9 vectors, without AP20187 intervention, were monitored to assess the exclusive impact of the vectors on tumor growth.

We then periodically assessed the tumor growth rate and volume up to day 10, as recommended earlier.[32] Figure 2B depicts the relative tumor growth pattern of the experimental mice. At day 10, the relative tumor volume (RTV) was significantly (~1.35-fold) reduced in AAV6 vector-treated groups when compared to mock-treated animals. The representative data from each experimental group are shown in Figure 2C. The harvested tumors from all five groups are presented in Figure S1. However, no significant difference was observed between AAV6 WT and AAV6 K31Q Neddylation mutant groups (AP20187 administered) *in vivo*, possibly due to the saturation of transgene expression, as has been observed in other studies.[33,34] There was a significant difference in RTV observed ($p \leq 0.001$) between AP20187 injected versus non-injected groups. Taken together, the AAV6-iCasp9 vector-mediated (WT and K31Q mutant) suicide gene therapy was well tolerated and significantly reduced breast cancer tumor growth *in vivo* in the presence of AP20187. However, a further increased dose of AAV6-iCasp9 vectors *via* a dose-finding study will be required to completely ablate the breast cancer tumor.

Histological analysis of AAV-treated breast tumor tissue correlates with the formation of apoptotic bodies

Breast tumors were harvested from the orthotropic mice 10 days after vector administration. For morphological analysis, the tumors were fixed, and paraffin sections were taken for H&E staining. Under 100× magnification, a comparison of cellular morphology was done. Compared to the mock control group, the AAV6-iCasp9-treated tumors (AP20187 administered) had an increased number of apoptotic cells with condensed nuclei (red arrow in Figure 3). Furthermore, the AP20187-treated animals also showed focal areas of necrosis. In contrast, the mock-treated and AAV6-iCasp9 vector-only administered group (without AP20187) had a higher distribution of pleomorphic nuclei (brown arrow), mitotic cells (black arrow), and dense nuclei with higher cell density were seen (Figure 3). Moreover, well-defined features such as cellular debris, shrunken cells, karyorrhectic (marked by yellow circle) and pyknotic nuclei were identified in

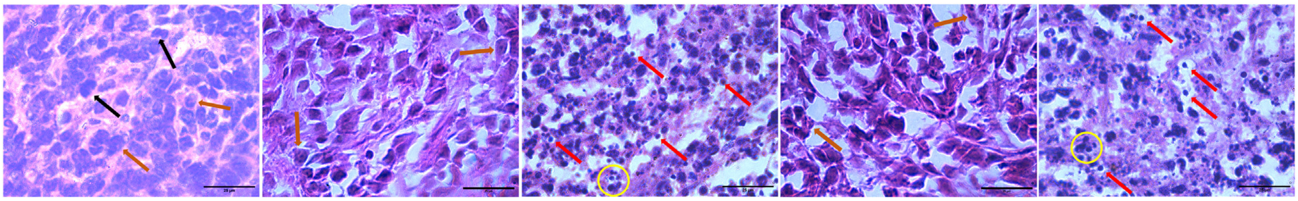

Figure 3. Histological analysis of tumors in breast cancer allograft tissue

Histological comparison among all of the groups depicted that AAV6-iCasp9 +AP20187-treated groups had focal areas of necrosis, pyknosis, karyorrhexis (marked by yellow circle), and apoptotic bodies (shown by red arrow) than the mock-treated and AAV6-iCasp9 vector-only groups (magnification 100×; scale bar, 25 μm), whereas more numbers of pleomorphic nuclei (marked by brown arrow) and mitotic cells (marked by black arrow) were observed in mock-treated and AAV6-iCasp9 vector-only groups. The data was generated from 2 representative tumors from each group and a total of 6 sections from each group.

the AAV6-iCasp9-treated groups (AP20187 administered) but not in the other experimental groups.[35] Similar findings have been reported for the *iCasp9*-based suicide gene therapy in an HCC murine model.[36]

AAV6-iCasp9 gene transfer activates the effector caspase in the apoptosis pathway

The major goal of the *iCasp9* gene therapy with AAV6 vectors is to trigger the apoptosis pathway. Breast tumors were explanted from all five groups and further homogenized; total RNA was isolated, and cDNA was synthesized. The induction of apoptosis was further confirmed by quantifying the *iCasp9* mRNA expression from the cDNA. A qPCR analysis showed that the relative expression of *iCasp9* was significantly increased by ∼6–37 times ($p \leq 0.001$) in all AAV6-iCasp9-treated groups (with or without AP20187) when compared to the mock-treated group (Figure S2). Further analysis showed that *iCasp9* expression was significantly ($p \leq 0.05$) high in the AAV6-iCasp9 capsid mutant-treated group when compared to the AAV6-WT vector-treated group. In addition, tumor sections of all of the AAV6-treated groups were stained with A20 antibody for the detection of intact AAV capsid, and the number of AAV^+ cells was quantified (Figure 4A). Figure 4B showed a significantly ($p \leq 0.05$) high number of AAV-transduced cells in the AAV6K31Q-CAG-iCasp9-treated group (39.6 ± 3.3) compared to the AAV6-CAG-iCasp9-treated group (28.8 ± 4.6). In addition, we found no detectable AAV^+ cells in the mock-treated group. The activation of cleaved caspase 3 in the breast tumor sections was then verified by immunohistochemistry (IHC). The cleaved caspase 3 expression was detected in both cytoplasm and nucleus, but the association of cleaved caspase 3 in the nuclei (represented by green arrow) of AAV6-iCasp9 + AP20187-treated groups was higher compared to other groups (Figure 5A). It must be noted that the cleavage and activation of caspase 3 are mediated by induced caspase 9,[37] and further nuclear translocation of cleaved caspase 3 initiates the apoptosis, as observed in our data (Figure 3).[38] Further quantification of the activation of cleaved caspase 3^+ showed that AAV6-iCasp9 vector-treated groups with AP20187 had significantly ($p \leq 0.001$) higher cleaved caspase 3 expression (AAV6-CAG-iCasp9: 21.8 ± 7.5; AAV6K31Q-CAG-iCasp9: 31.6 ± 15.2) when compared to mock-treated and AAV6-iCasp9 vector-only groups (Figure 5B). This signifies that AAV6-iCasp9 vectors failed to activate the caspase 3 in the absence of AP20187, and overexpressed *iCasp9* showed functional activity only in the presence of this prodrug.

DNA damage correlates with treatment outcomes in breast tumor

To evaluate the molecular effects of *iCasp9*, we also performed a terminal deoxynucleotidyl transferase–mediated dUTP nick-end labeling test (TUNEL). TUNEL assay can detect double-strand breaks in the DNA of apoptotic cells and thus is used as a biomarker for the diagnosis of cell death.[39] Tissues from both AAV6-iCasp9-treated groups in the presence of AP20187 were strikingly positive for $TUNEL^+$ cells, indicating widespread cell death, which is absent in the AAV6-iCasp9 vector-only group (Figure S3A). To quantify DNA damage, we assessed the fluorescence-integrated density of $TUNEL^+$ cells. Data depicted in Figure S3B reveals $TUNEL^+$ cells with higher fluorescence-integrated density in tumors that received suicide gene therapy (AAV6-CAG-iCasp9: 1.7×10^5 and AAV6K31Q-CAG-iCasp9: 2×10^5 per unit area, $p \leq 0.05$ and $p \leq 0.001$, respectively) when compared to the mock-treated group. These data highlight that the AAV6K31Q mutant is effective in triggering apoptosis in the breast cancer cells in the presence of AP20187. However, a number of factors, including vector saturation dose, where a dose of $\sim 5 \times 10^{10}$ vg/animal may be close to the saturation levels and which may have contributed to the similar regression pattern found in the WT and mutant vector–treated animals.[34,40–42] Although we noticed high AAV transduction in the mutant group, the same does not get reflected in terms of RTV, and we speculate that this discrepancy may stem from the limited availability of the dimerizer drug AP20187, which is integral to the ultimate cell kill effect. Future investigations focusing on dose optimization of AP20187 could help elucidate its impact on RTV.

To assess the off-target effect of *iCasp9* activation in normal organs (e.g., liver) of AAV6-iCasp9-treated animals, IHC of caspase 9 protein was performed. Figure S4 demonstrated the absence of caspase 9 expression in the liver of AAV vector–administered groups. This

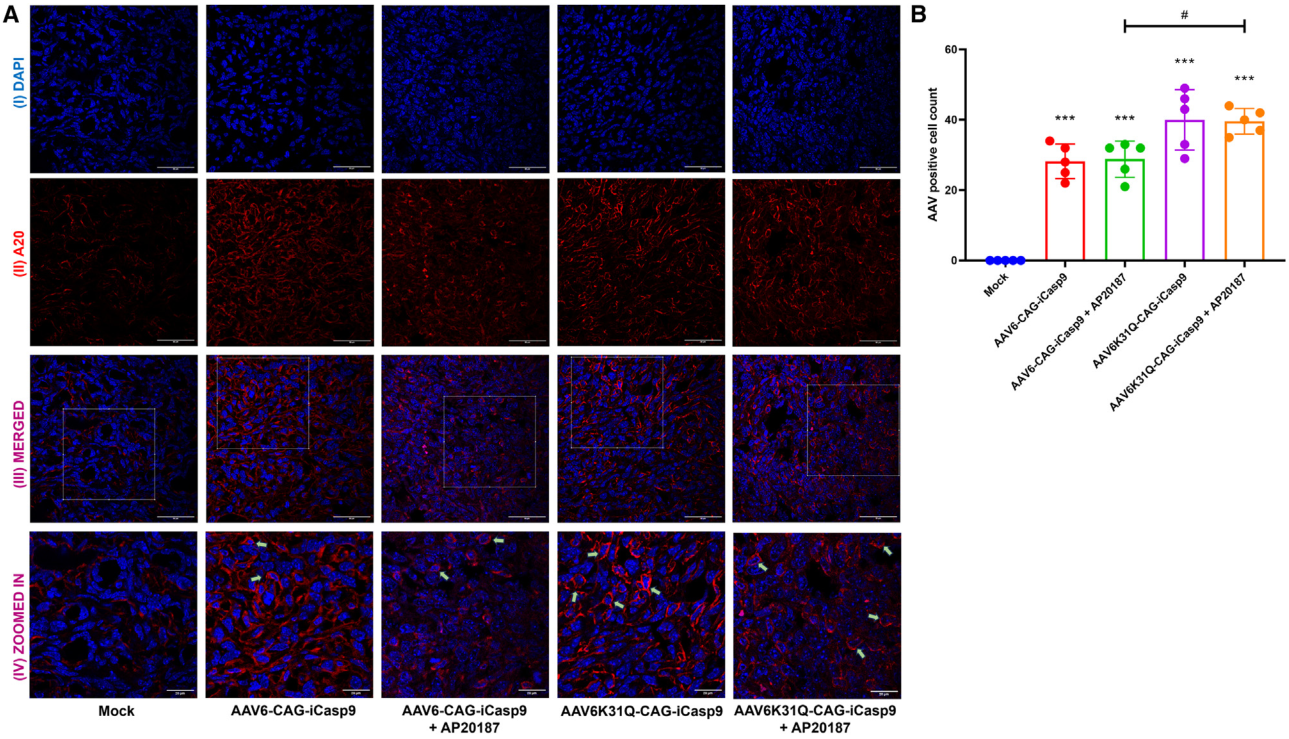

Figure 4. Detection of AAV particles in breast cancer tissue

Tumors from each treatment group were analyzed by IHC with A20 antibody specific to detect AAV capsid. (A) Micrographs from the AAV-treated groups with or without AP20187 had a clear distribution of AAV^+ cells within the tumors when compared to mock-treated animals (III, magnification 63×; scale bar, 50 μm). The magnified regions of (III) are represented in (IV) (scale bar, 25 μm). AAV^+ cells are marked with green arrows. (B) Quantification using ImageJ showed that the number of AAV^+ cells was higher in the AAV6K31Q-CAG-iCasp9-treated group compared to the AAV6-CAG-iCasp9-treated group (***$p \leq 0.001$ versus mock group; $^{\#}p \leq 0.05$ versus AAV6-CAG-iCasp9 + AP20187). Data are represented as mean ± SD. The data are generated from 5 sections from each treatment group.

implies that this direct intratumoral suicide gene therapy had a negligible effect on normal tissues.

DISCUSSION

TNBC has an increased resistance against available chemotherapies such as taxane and anthracycline-based drugs, emphasizing the critical need for research into novel strategies to slow disease progression.[43,44] Gene therapy, by virtue of its direct mechanism of triggering apoptosis in the target cell, is an effective approach for cancer treatment.[45] In particular, gene therapy using an AAV vector in breast cancer has shown encouraging results. A study was conducted in the NeuT breast cancer model with a septuplet-tyrosine mutant form of AAV2 vector-mediated delivery of small interfering RNAs (siRNAs) against proteins involved in unfolded protein response.[46] The outcome data showed that treated xenograft tumors had decreased growth and angiogenesis. In another study, suicide gene therapy by intratumoral administration of doxycycline-inducible AAV2-HSV-TK resulted in substantial suppression of tumor growth in a mouse model of breast cancer (MCF-7).[10] In a study of immunomodulation of neu-expressing Turin-Bologna breast cancer tumors, AAV5-neu or AAV6-neu expression increased the survival (80% versus 100%, up to day 300) of the animal model by inducing long-term immunity against the tumor.[27] Pinto et al. showed that the transduction of MDA-MB-468 cells with an AAV2-short hairpin RNA vector directed against proteasome subunit alpha-2 caused a dramatic reduction in cell survivability (from 100% to 30%) and an increase in the induction of apoptosis, by a factor of two.[47] AAV2-mediated overexpression of high-affinity vascular endothelial growth factor blocker and paclitaxel improved therapeutic response in a murine model of TNBC.[48] Here, for the first time, we have demonstrated the therapeutic potency of the *iCasp9* transgene in the murine TNBC model.[49]

The inducible caspase 9-based system offers several benefits compared to enzyme-activated prodrug therapies, as highlighted earlier.[20] First, iCasp9/AP20187-based suicide gene effects have an acute onset of activity and functional rescue in terms of days (6–10 days versus 30 days) in animal models compared to other enzyme-activated prodrug therapies.[9,20,36,50–52] Second, the cell-cycle dependency of enzyme-activated prodrug therapies limits their cytotoxic effects to the actively dividing cells only, which may decrease their therapeutic potential in a heterogeneous tumor tissue consisting of senescence cancer stem cells.[53,54] Upon the administration of AAV6-iCasp9 vectors, caspase 3 and other components of the intrinsic apoptotic pathway are activated after the dimerization of

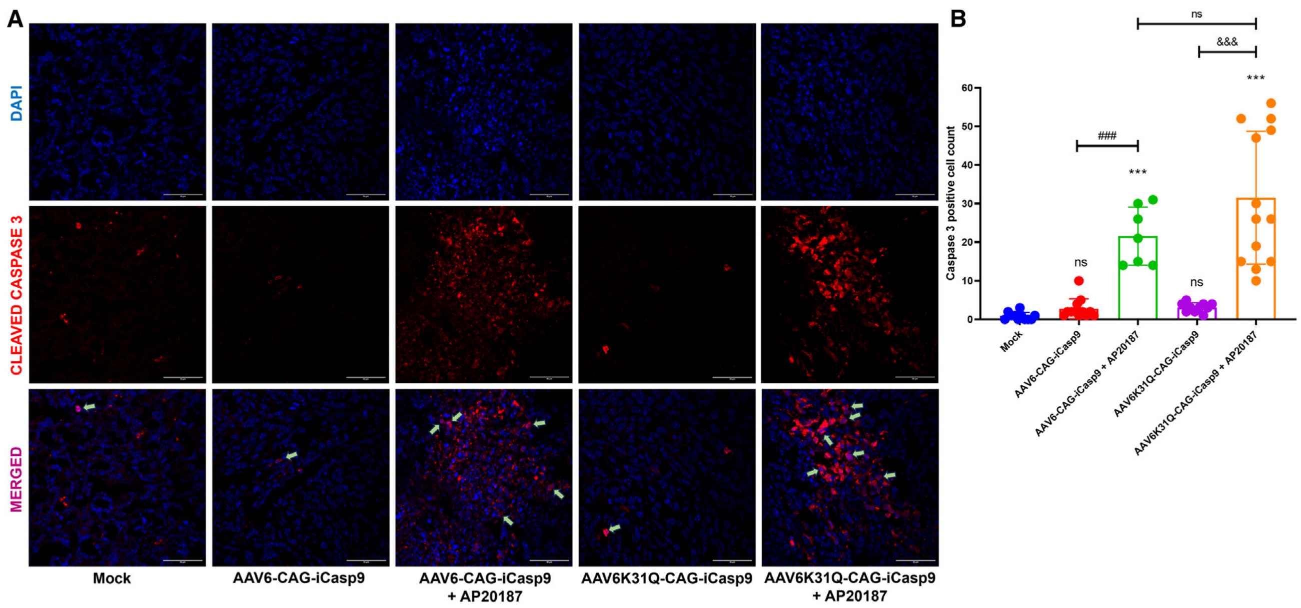

Figure 5. Detection of activated caspase 3 in breast cancer tissue

After receiving AAV6-iCasp9 gene therapy, breast tumors were harvested, and IHC was performed after fixation and sectioning of tumors. (A) Micrographs representing the treatment groups (AAV6-CAG-iCasp9 and AAV6K31Q-CAG-iCasp9) with AP20187 showed enhanced association of cleaved caspase 3 in the nuclei compared to mock-treated animals (represented by green arrows; magnification 63×; scale bar, 50 μm). AAV6-CAG-iCasp9 and AAV6K31Q-CAG-iCasp9 without AP20187 failed to activate the caspase 3 protein downstream of caspase 9. (B) The number of caspase 3^{+} cells was quantified using ImageJ software (***$p \leq 0.001$; ns [not significant] versus mock group; ###$p \leq 0.001$ versus AAV6-CAG-iCasp9; &&& $p \leq 0.001$ versus AAV6K31Q-CAG-iCasp9). Data are represented as mean ± SD. The data were generated from 7 to 13 sections from each treatment group.

iCasp9 protein by AP20187.[55,56] This activation of the proapoptotic pathway occurs directly and is not dependent on cell-cycle phase.[57] Our *in vivo* study has demonstrated that after administering the AAV6-iCasp9 vector/AP20187 combination, the breast tumors had a lesser tumor volume and growth rate within 6–10 days of vector administration. In comparison, previous studies in breast cancer models showed retarded tumor growth after 30 days of administration of HSV-TK/ganciclovir-based gene therapy.[10] The induction of apoptosis was also further validated by *ex vivo* analysis of breast tumor tissue. Although we have observed the therapeutic potential of our *iCasp9* gene at the cellular level, despite having breast tumor growth retardation, tumors were not completely eradicated. This suggests that monotherapy may not be sufficient, and further enhancement by using an adjunct or combinatorial treatment with chemotherapeutic or immunotherapeutic drugs is required.

Our study has the following limitations. We have used a strong ubiquitous CAG promoter for the expression of *iCasp9* transgene. The therapy will be more beneficial if further tumor-targeting strategies at the transcription or vector transduction levels can be applied. Instead of using ubiquitous promoters, using a cancer-specific or tumor-specific promoter is desirable.[58,59] Yun et al. have shown that promoters of the protein regulator of cytokinesis 1 (*PRC1*) and ribonuclease reductase 2 (*RRM2*) genes can be used in targeting breast cancer cells because these promoters exhibit strong gene expression similar to that driven by the CMV promoter.[60] At the transduction level, the AAV vector can be modified by peptide insertion and then targeted towards specific cancer cells. A previous study from our lab showed a modified AAV6 vector carrying CD33 targeting peptide against human myeloid leukemia cells (U937).[21] Another strategy to augment the effectivity of *iCasp9*-based gene therapy could be the modulation of hallmark parameters of the tumor microenvironment, such as hypoxia, known to confer resistance to certain forms of therapy.[61] Therefore, enhancing the *iCasp9* system by altering the regulatory components of the transgenic construct, increasing the effective vector dose from the currently used $\sim 5 \times 10^{10}$ vg by a log fold, or combining it with other adjunct therapies may pave the way for its clinical translation in patients with breast cancer.

MATERIALS AND METHODS

Cell culture

The mouse mammary carcinoma (4T1) cell line was a gift from Dr. Dipak Datta, Central Drug Research Institute, Lucknow, India. The AAV-293 cell line was purchased from Stratagene (San Diego, CA). Cell lines were maintained at 37°C and 5% CO_2 in a growth medium containing Iscove's modified Dulbecco's medium (IMDM) supplemented with 10% fetal bovine serum (Gibco, Waltham, MA), 10 μg/mL each of ciprofloxacin (HiMedia Laboratories, Mumbai, India), and piperacillin (MP Biomedicals, Santa Ana, CA). B/B homodimerizer or AP20187 was purchased from ARIAD Pharmaceuticals

(Cambridge, MA) and was reconstituted in 100% ethanol at a 1-mM concentration and kept at −20°C.

Prediction of Neddylation sites on AAV6 capsid

Amino acids for Neddylation were predicted in the VP1 protein of AAV6 using the sequence of the VP1 protein (Protein ID: AAB95450.1). NeddyPreddy, a web-based bioinformatics tool, was used to predict potential sites for Neddylation.[62] The output was used to determine the medium and high threshold levels for this tool. The AAV6 K31Q target was selected based on the high threshold score of 0.9 out of 1. The Neddylation mutation (K31Q) in the AAV6 WT rep/cap plasmid (p.AAVR2/C6) was further generated by site-directed mutagenesis using specific primers.

Generation of recombinant AAV vector

The AAV6 capsid K31 residue was changed to glutamine (K > Q) using a QuikChange II XL Site-Directed Mutagenesis Kit (Agilent Technologies, Santa Clara, CA) according to the manufacturer's instructions. Triple transfection was performed to generate recombinant AAV vectors in the AAV-293 cell line. We used 15 cm^2 dishes to grow AAV-293 cells until they reached 80% confluency. The AAV6 WT or Neddylation mutant (K31Q) rep/cap plasmid (p.AAVR2/C6), *iCasp9* transgene under CAG promoter (p.AAV-CAG-iCasp9), and adenoviral helper plasmids (p.Helper) were cotransfected into the AAV-293 cells in the presence of serum-free IMDM media using equimolar polyethyleneimine (Polysciences, Warrington, PA).[63] Cells were harvested after 68–72 h, lysed followed by benzonase (25 U/mL; Sigma-Aldrich, St. Louis, MO) treatment. For the purification of vectors, the following steps were followed: iodixanol gradient ultracentrifugation (OptiPrep, Sigma-Aldrich) and column chromatography (Cytiva, Marlborough, MA). Finally, the concentration of the purified vectors up to 500 μL was done using Amicon Ultra-15 centrifugal filtration device (Millipore, Bedford, MA).

Titration for quantification of AAV vector genome

The physical particle titer of AAV vectors was measured. The sample was treated with DNase to remove free DNA. A qPCR with SYBR Green (Promega, Madison, WI) and polyadenylation (PolyA) signal-specific primer was carried out in CFX96 (Bio-Rad, Hercules, CA). AAV2-RSS (American Type Culture Collection, Manassas, VA) was used as a standard for titration. Titers were measured in vg/mL and calculated from two independent analyses.

In vitro cytotoxicity assay

For the comparison of cytotoxicity by AAV6-CAG-iCasp9 and AAV6K31Q-CAG-iCasp9 vectors, $\sim 1.5 \times 10^4$ 4T1 cells were seeded in triplicate and transduced at MOIs of 5×10^3, 5×10^4, and 1×10^5. Vector-transduced cells were treated with 10 nM AP20187 the next day. An absorbance-based EZcountTM MTT Cell Assay Kit (HiMedia, Mumbai, India) was used to evaluate cell cytotoxicity 24 h after drug treatment. The percentage of cell survivability was calculated as described by the manufacturer protocol.[10]

Inducible *Casp9* gene therapy in breast cancer murine model

Female athymic$^{nu/nu}$ mice (National Institute of Nutrition, Hyderabad, India) were used for this study. Animal experiments were conducted following the approval of the IIT-Kanpur institutional animal ethics committee. For orthotopic injection in 6- to 8-week-old female athymic$^{nu/nu}$ mice, $\sim 1 \times 10^6$ 4T1 cells were resuspended in 100 μL filtered PBS and administered in the sixth mammary fat pad region. Upon reaching a tumor volume of ∼100–150 mm^3, mice received $\sim 5 \times 10^{10}$ vg of AAV6-CAG-iCasp9 or AAV6K31Q-CAG-iCasp9 vectors intratumorally.[20] Approximately 0.1 mL of PBS was injected to mimic the injection process in the mock group. After administering the vector, the animals received 3 doses of intraperitoneal AP20187 (1 mg/kg) injections at 48-h intervals starting the next day of vector administration. The control groups included two treatment groups that only received the AAV6-CAG-iCasp9 or AAV6K31Q-CAG-iCasp9 vectors and a third group receiving the sham intraperitoneal injection to observe the AAV6-iCasp9 vector-only effect in the tumor development. Tumor diameter was measured every alternate day using a Vernier caliper at two perpendicular diameters in the experimental mice. The animals were examined visually for any other gross changes daily. Tumor volume was calculated using the equation $0.5 \times L \times W^2$, where L and W are the longest and the smallest diameter (mm), respectively.[64] RTV was calculated by dividing tumor volume at a particular day (T_X) by tumor volume at day 0 (T_0), where RTV_T = RTV of the treated group and RTV_M = RTV of the mock group.[65]

Gene expression quantification

Total RNA was isolated from the breast tumor tissue of each group. Approximately 50 mg of tissue was first homogenized using IG-L13K Mini Handheld Homogenizer (iGene Labserve, New Delhi, India), and further RNA isolation was done with Trizol (Invitrogen, Waltham, MA) reagent. Isolated RNA was run in a 1.5% agarose gel (with 1% sodium hypochlorite), and the integrity of 28S, 18S, and 5S rRNA was checked. For analysis of *iCasp9* mRNA expression, a qPCR assay with SYBR Green (Promega) was performed (3–6 technical replicates per group). The primer sequence was 5′- GAAGGG GTTGCCCAGATGAG-3′ (forward) and 5′- GCACCGACATCACC AAATCC-3′ (reverse). The relative normalized expression of *iCasp9* was plotted using 18S rRNA expression as a reference for each sample.

Histopathological studies

Tumors from mock and AAV-treated groups were harvested after 10 days of AAV vector administration, washed in PBS, and fixed in 10% neutral buffered formalin at room temperature for 24 h. Tumor tissues were further processed by paraffin embedding, tissue sectioning, and H&E staining. Thereafter, stained sections were analyzed by microscopic examination.[66] Images were acquired under an inverted microscope (Leica DMi8, Leica Microsystems, Wetzlar, Germany) at 100× magnification.

In situ cell death detection

Paraffin blocks containing tumor tissue were cut using a microtome (Leica Biosystems) to obtain tumor tissue sections. Following this,

the 8-μm sections (n = 6–8) were processed through a TUNEL assay following the manufacturer's protocol (Roche, Basel, Switzerland) to detect cells undergoing DNA damage and apoptosis.[39] Additional washing steps were followed by nuclear staining with DAPI (1:1,000; Thermo Fisher, Waltham, MA) and mounting (FluorSave Reagent; Millipore, Merck, Burlington, MA). Imaging was performed using a confocal microscope (LSM780NLO, Carl Zeiss GmbH, Wein, Austria). Micrographs were acquired at a 40× magnification. The fluorescence-integrated density was measured using ImageJ (NIH, Bethesda, MD) software.[67]

IHC

For the IHC assay to evaluate the level of AAV and cleaved caspase 3, the tumor tissue was fixed and processed according to standard protocols. Furthermore, tumor and liver tissues were sectioned at 6- and 8-μm thickness using a cryostat (Leica CM1250, Leica Biosystems, Wetzlar, Germany), respectively. Thereafter, tissue sections were again fixed in 4% paraformaldehyde and staining was carried out with mouse anti-caspase 9 (1:150, sc-56076, Santa Cruz, Dallas, TX) or mouse anti-AAV (A20; 1:100, 10R-A110A Fitzgerald, Biosynth, Staad, Switzerland) or rabbit anti-cleaved caspase 3 antibody [1:200, 9664S, Cell Signaling Technology, Danvers, MA) to indicate the activation of the apoptotic pathway downstream of activated caspase 9.[68] Alexa Fluor 568 goat anti-mouse immunoglobulin G (IgG) (1:250, Thermo Fisher) was used as the secondary antibody for anti-caspase 9 and A20. Cy3 AffiniPure goat anti-rabbit IgG (1:200, Jackson ImmunoResearch, West Grove, PA) was used as the secondary antibody for anti-cleaved caspase 3. Finally, after washing, sections were counterstained with DAPI (1:1,000) and mounted with mounting media. Micrographs were acquired from a confocal microscope (LSM780NLO, Carl Zeiss GmbH), and AAV or cleaved caspase 3^+ cells were counted using ImageJ software.

Statistical analysis

Statistical significance was evaluated by an unpaired t test with Welch's correction or a two-way ANOVA with Bonferroni correction, as appropriate (GraphPad Prism 8.0 Software, La Jolla, CA).

DATA AND CODE AVAILABILITY

The data presented in the manuscript will be available upon reasonable request to the corresponding author.

SUPPLEMENTAL INFORMATION

Supplemental information can be found online at https://doi.org/10.1016/j.omtm.2023.101166.

ACKNOWLEDGMENTS

We thank Central Experimental Animal Facility, IIT Kanpur, for housing the animals and providing the microtome and cryostat facilities for tissue sectioning; Live Cell Imaging Lab, ACMS, IIT Kanpur, for assistance during confocal imaging; Dr. Saraswat's Pathology, Kanpur for performing paraffin embedding and H&E staining; Mr. Mohankumar B. Senthilkumar for helping with the IHC experiments. We thank Dr. Dipak Datta, Central Drug Research Institute, Lucknow, India, for providing the 4T1 cells and guidance for the generation of the animal model. This research work has been supported by the intramural fund from IIT Kanpur. S.P. and V.S. received study fellowships from the Human Resource Development Group, Council of Scientific & Industrial Research and the Ministry of Human Resource Development, Government of India, respectively.

AUTHOR CONTRIBUTIONS

S.P., V.S., and N.K. performed the experiments; S.P. and G.R.J. interpreted the data and wrote the manuscript; G.R.J. conceptualized and designed the study.

DECLARATION OF INTERESTS

S.P., V.S., N.K., and G.R.J. have applied for patents based on AAV, and some of these have been licensed to commercial entities.

REFERENCES

1. Harbeck, N., Penault-Llorca, F., Cortes, J., Gnant, M., Houssami, N., Poortmans, P., Ruddy, K., Tsang, J., and Cardoso, F. (2019). Breast cancer. Nat. Rev. Dis. Prim. *5*, 66.
2. Sung, H., Ferlay, J., Siegel, R.L., Laversanne, M., Soerjomataram, I., Jemal, A., and Bray, F. (2021). Global Cancer Statistics 2020: GLOBOCAN Estimates of Incidence and Mortality Worldwide for 36 Cancers in 185 Countries. CA A Cancer J. Clin. *71*, 209–249.
3. Denkert, C., Liedtke, C., Tutt, A., and von Minckwitz, G. (2017). Molecular alterations in triple-negative breast cancer-the road to new treatment strategies. Lancet *389*, 2430–2442.
4. Ji, X., Lu, Y., Tian, H., Meng, X., Wei, M., and Cho, W.C. (2019). Chemoresistance mechanisms of breast cancer and their countermeasures. Biomed. Pharmacother. *114*, 108800.
5. Dastjerd, N.T., Valibeik, A., Rahimi Monfared, S., Goodarzi, G., Moradi Sarabi, M., Hajabdollahi, F., Maniati, M., Amri, J., and Samavarchi Tehrani, S. (2022). Gene therapy: A promising approach for breast cancer treatment. Cell Biochem. Funct. *40*, 28–48.
6. Belete, T.M. (2021). The Current Status of Gene Therapy for the Treatment of Cancer. Biologics. *15*, 67–77.
7. Zarogoulidis, P., Darwiche, K., Sakkas, A., Yarmus, L., Huang, H., Li, Q., Freitag, L., Zarogoulidis, K., and Malecki, M. (2013). Suicide Gene Therapy for Cancer - Current Strategies. J. Genet. Syndr. Gene Ther. *4*, 16849.
8. Singh, V., Khan, N., and Jayandharan, G.R. (2022). Vector engineering, strategies and targets in cancer gene therapy. Cancer Gene Ther. *29*, 402–417.
9. Li, L.Q., Shen, F., Xu, X.Y., Zhang, H., Yang, X.F., and Liu, W.G. (2013). Gene therapy with HSV1-sr39TK/GCV exhibits a stronger therapeutic efficacy than HSV1-TK/GCV in rat C6 glioma cells. Sci. World J. *2013*, 951343.
10. Li, Z.B., Zeng, Z.J., Chen, Q., Luo, S.Q., and Hu, W.X. (2006). Recombinant AAV-mediated HSVtk gene transfer with direct intratumoral injections and Tet-On regulation for implanted human breast cancer. BMC Cancer *6*, 66.
11. Emamian, M., Abbaspour, A., Shahani, T., Biglari, A., and Sharafi, A. (2021). Non-viral Suicide Gene Therapy: Cytosine Deaminase Gene Directed by VEGF Promoter and 5-fluorocytosine as a Gene Directed Enzyme/prodrug System in Breast Cancer Model. Drug Res. *71*, 395–406.
12. Zhang, J., Kale, V., and Chen, M. (2015). Gene-directed enzyme prodrug therapy. AAPS J. *17*, 102–110.
13. Straathof, K.C., Pulè, M.A., Yotnda, P., Dotti, G., Vanin, E.F., Brenner, M.K., Heslop, H.E., Spencer, D.M., and Rooney, C.M. (2005). An inducible caspase 9 safety switch for T-cell therapy. Blood *105*, 4247–4254.
14. Di Stasi, A., Tey, S.K., Dotti, G., Fujita, Y., Kennedy-Nasser, A., Martinez, C., Straathof, K., Liu, E., Durett, A.G., Grilley, B., et al. (2011). Inducible apoptosis as a safety switch for adoptive cell therapy. N. Engl. J. Med. *365*, 1673–1683.

15. Nakashima, I., Saito, S., Akahoshi, E., Yagyu, S., Sugano-Ishihara, M., and Nakazawa, Y. (2022). Non-viral inducible caspase 9 mRNA delivery using lipid nanoparticles against breast cancer: An *in vitro* study. Biochem. Biophys. Res. Commun. *635*, 144–153.

16. Wang, D., Tai, P.W.L., and Gao, G. (2019). Adeno-associated virus vector as a platform for gene therapy delivery. Nat. Rev. Drug Discov. *18*, 358–378.

17. Luo, J., Luo, Y., Sun, J., Zhou, Y., Zhang, Y., and Yang, X. (2015). Adeno-associated virus-mediated cancer gene therapy: current status. Cancer Lett. *356*, 347–356.

18. Balakrishnan, B., and Jayandharan, G.R. (2014). Basic biology of adeno-associated virus (AAV) vectors used in gene therapy. Curr. Gene Ther. *14*, 86–100.

19. Khan, N., Mahajan, N.K., Sinha, P., and Jayandharan, G.R. (2019). An efficient method to generate xenograft tumor models of acute myeloid leukemia and hepatocellular carcinoma in adult zebrafish. Blood Cells Mol. Dis. *75*, 48–55.

20. Khan, N., Bammidi, S., Chattopadhyay, S., and Jayandharan, G.R. (2019). Combination Suicide Gene Delivery with an Adeno-Associated Virus Vector Encoding Inducible Caspase-9 and a Chemical Inducer of Dimerization Is Effective in a Xenotransplantation Model of Hepatocellular Carcinoma. Bioconjugate Chem. *30*, 1754–1762.

21. Khan, N., Bammidi, S., and Jayandharan, G.R. (2019). A CD33 Antigen-Targeted AAV6 Vector Expressing an Inducible Caspase-9 Suicide Gene Is Therapeutic in a Xenotransplantation Model of Acute Myeloid Leukemia. Bioconjugate Chem. *30*, 2404–2416.

22. Yan, Z., Zak, R., Luxton, G.W.G., Ritchie, T.C., Bantel-Schaal, U., and Engelhardt, J.F. (2002). Ubiquitination of both adeno-associated virus type 2 and 5 capsid proteins affects the transduction efficiency of recombinant vectors. J. Virol. *76*, 2043–2053.

23. Kamitani, T., Kito, K., Nguyen, H.P., and Yeh, E.T. (1997). Characterization of NEDD8, a developmentally down-regulated ubiquitin-like protein. J. Biol. Chem. *272*, 28557–28562.

24. Zhang, X., Ye, Z., Pei, Y., Qiu, G., Wang, Q., Xu, Y., Shen, B., and Zhang, J. (2016). Neddylation is required for herpes simplex virus type I (HSV-1)-induced early phase interferon-beta production. Cell. Mol. Immunol. *13*, 578–583.

25. Maurya, S., Mary, B., and Jayandharan, G.R. (2019). Rational Engineering and Preclinical Evaluation of Neddylation and SUMOylation Site Modified Adeno-Associated Virus Vectors in Murine Models of Hemophilia B and Leber Congenital Amaurosis. Hum. Gene Ther. *30*, 1461–1476.

26. Maurya, S., Mary, B., and Jayandharan, G.R. (2020). Improved ocular gene transfer with a Neddylation-site modified AAV-RPE65 vector in rd12 mice. Eye *34*, 1313–1315.

27. Steel, J.C., Di Pasquale, G., Ramlogan, C.A., Patel, V., Chiorini, J.A., and Morris, J.C. (2013). Oral vaccination with adeno-associated virus vectors expressing the Neu oncogene inhibits the growth of murine breast cancer. Mol. Ther. *21*, 680–687.

28. Sayroo, R., Nolasco, D., Yin, Z., Colon-Cortes, Y., Pandya, M., Ling, C., and Aslanidi, G. (2016). Development of novel AAV serotype 6 based vectors with selective tropism for human cancer cells. Gene Ther. *23*, 18–25.

29. Aurnhammer, C., Haase, M., Muether, N., Hausl, M., Rauschhuber, C., Huber, I., Nitschko, H., Busch, U., Sing, A., Ehrhardt, A., and Baiker, A. (2012). Universal real-time PCR for the detection and quantification of adeno-associated virus serotype 2-derived inverted terminal repeat sequences. Hum. Gene Ther. Methods *23*, 18–28.

30. Pulaski, B.A., and Ostrand-Rosenberg, S. (2001). Mouse 4T1 breast tumor model. Curr. Protoc. Im. *20*, 20.2.

31. Yagyu, S., Hoyos, V., Del Bufalo, F., and Brenner, M.K. (2015). An Inducible Caspase-9 Suicide Gene to Improve the Safety of Therapy Using Human Induced Pluripotent Stem Cells. Mol. Ther. *23*, 1475–1485.

32. Sápi, J., Kovács, L., Drexler, D.A., Kocsis, P., Gajári, D., and Sápi, Z. (2015). Tumor Volume Estimation and Quasi-Continuous Administration for Most Effective Bevacizumab Therapy. PLoS One *10*, e0142190.

33. Wu, T., Töpfer, K., Lin, S.W., Li, H., Bian, A., Zhou, X.Y., High, K.A., and Ertl, H.C.J. (2012). Self-complementary AAVs induce more potent transgene product-specific immune responses compared to a single-stranded genome. Mol. Ther. *20*, 572–579.

34. Petrs-Silva, H., Dinculescu, A., Li, Q., Min, S.H., Chiodo, V., Pang, J.J., Zhong, L., Zolotukhin, S., Srivastava, A., Lewin, A.S., and Hauswirth, W.W. (2009). High-efficiency transduction of the mouse retina by tyrosine-mutant AAV serotype vectors. Mol. Ther. *17*, 463–471.

35. Elmore, S.A., Dixon, D., Hailey, J.R., Harada, T., Herbert, R.A., Maronpot, R.R., Nolte, T., Rehg, J.E., Rittinghausen, S., Rosol, T.J., et al. (2016). Recommendations from the INHAND Apoptosis/Necrosis Working Group. Toxicol. Pathol. *44*, 173–188.

36. Khan, N., Maurya, S., Bammidi, S., and Jayandharan, G.R. (2020). AAV6 Vexosomes Mediate Robust Suicide Gene Delivery in a Murine Model of Hepatocellular Carcinoma. Mol. Ther. Methods Clin. Dev. *17*, 497–504.

37. Riedl, S.J., and Shi, Y. (2004). Molecular mechanisms of caspase regulation during apoptosis. Nat. Rev. Mol. Cell Biol. *5*, 897–907.

38. Kamada, S., Kikkawa, U., Tsujimoto, Y., and Hunter, T. (2005). Nuclear translocation of caspase-3 is dependent on its proteolytic activation and recognition of a substrate-like protein(s). J. Biol. Chem. *280*, 857–860.

39. Kyrylkova, K., Kyryachenko, S., Leid, M., and Kioussi, C. (2012). Detection of apoptosis by TUNEL assay. Methods Mol. Biol. *887*, 41–47.

40. Aronson, S.J., Bakker, R.S., Moenis, S., van Dijk, R., Bortolussi, G., Collaud, F., Shi, X., Duijst, S., Ten Bloemendaal, L., Ronzitti, G., et al. (2020). A Quantitative In Vitro Potency Assay for Adeno-Associated Virus Vectors Encoding for the UGT1A1 Transgene. Mol. Ther. Methods Clin. Dev. *18*, 250–258.

41. Hakim, C.H., Clément, N., Wasala, L.P., Yang, H.T., Yue, Y., Zhang, K., Kodippili, K., Adamson-Small, L., Pan, X., Schneider, J.S., et al. (2020). Micro-dystrophin AAV Vectors Made by Transient Transfection and Herpesvirus System Are Equally Potent in Treating mdx Mouse Muscle Disease. Mol. Ther. Methods Clin. Dev. *18*, 664–678.

42. Welles, H.C., Jennewein, M.F., Mason, R.D., Narpala, S., Wang, L., Cheng, C., Zhang, Y., Todd, J.P., Lifson, J.D., Balazs, A.B., et al. (2018). Vectored delivery of anti-SIV envelope targeting mAb via AAV8 protects rhesus macaques from repeated limiting dose intrarectal swarm SIVsmE660 challenge. PLoS Pathog. *14*, e1007395.

43. Bai, X., Ni, J., Beretov, J., Graham, P., and Li, Y. (2021). Triple-negative breast cancer therapeutic resistance: Where is the Achilles' heel? Cancer Lett. *497*, 100–111.

44. Nedeljkovic, M., and Damjanovic, A. (2019). Mechanisms of Chemotherapy Resistance in Triple-Negative Breast Cancer-How We Can Rise to the Challenge. Cells *8*.

45. Sun, W., Shi, Q., Zhang, H., Yang, K., Ke, Y., Wang, Y., and Qiao, L. (2019). Advances in the techniques and methodologies of cancer gene therapy. Discov. Med. *27*, 45–55.

46. Ruan, Q., Xi, L., Boye, S.L., Han, S., Chen, Z.J., Hauswirth, W.W., Lewin, A.S., Boulton, M.E., Law, B.K., Jiang, W.G., et al. (2013). Development of an anti-angiogenic therapeutic model combining scAAV2-delivered siRNAs and noninvasive photoacoustic imaging of tumor vasculature development. Cancer Lett. *332*, 120–129.

47. Pinto, C., Silva, G., Ribeiro, A.S., Oliveira, M., Garrido, M., Bandeira, V.S., Nascimento, A., Coroadinha, A.S., Peixoto, C., Barbas, A., et al. (2019). Evaluation of AAV-mediated delivery of shRNA to target basal-like breast cancer genetic vulnerabilities. J. Biotechnol. *300*, 70–77.

48. Lu, L., Luo, S.T., Shi, H.S., Li, M., Zhang, H.L., He, S.S., Liu, Y., Pan, Y., and Yang, L. (2012). AAV2-mediated gene transfer of VEGF-Trap with potent suppression of primary breast tumor growth and spontaneous pulmonary metastases by long-term expression. Oncol. Rep. *28*, 1332–1338.

49. Kaur, P., Nagaraja, G.M., Zheng, H., Gizachew, D., Galukande, M., Krishnan, S., and Asea, A. (2012). A mouse model for triple-negative breast cancer tumor-initiating cells (TNBC-TICs) exhibits similar aggressive phenotype to the human disease. BMC Cancer *12*, 120.

50. Wang, Y.G., Huang, P.P., Zhang, R., Ma, B.Y., Zhou, X.M., and Sun, Y.F. (2016). Targeting adeno-associated virus and adenoviral gene therapy for hepatocellular carcinoma. World J. Gastroenterol. *22*, 326–337.

51. Torimura, T., Ueno, T., Taniguchi, E., Masuda, H., Iwamoto, H., Nakamura, T., Inoue, K., Hashimoto, O., Abe, M., Koga, H., et al. (2012). Interaction of endothelial progenitor cells expressing cytosine deaminase in tumor tissues and 5-fluorocytosine administration suppresses growth of 5-fluorouracil-sensitive liver cancer in mice. Cancer Sci. *103*, 542–548.

52. Kanazawa, T., Mizukami, H., Okada, T., Hanazono, Y., Kume, A., Nishino, H., Takeuchi, K., Kitamura, K., Ichimura, K., and Ozawa, K. (2003). Suicide gene therapy

using AAV-HSVtk/ganciclovir in combination with irradiation results in regression of human head and neck cancer xenografts in nude mice. Gene Ther. *10*, 51–58.

53. Kawashita, Y., Ohtsuru, A., Kaneda, Y., Nagayama, Y., Kawazoe, Y., Eguchi, S., Kuroda, H., Fujioka, H., Ito, M., Kanematsu, T., and Yamashita, S. (1999). Regression of hepatocellular carcinoma *in vitro* and *in vivo* by radiosensitizing suicide gene therapy under the inducible and spatial control of radiation. Hum. Gene Ther. *10*, 1509–1519.

54. Walcher, L., Kistenmacher, A.K., Suo, H., Kitte, R., Dluczek, S., Strauß, A., Blaudszun, A.R., Yevsa, T., Fricke, S., and Kossatz-Boehlert, U. (2020). Cancer Stem Cells-Origins and Biomarkers: Perspectives for Targeted Personalized Therapies. Front. Immunol. *11*, 1280.

55. Rossignoli, F., Grisendi, G., Spano, C., Golinelli, G., Recchia, A., Rovesti, G., Orsi, G., Veronesi, E., Horwitz, E.M., and Dominici, M. (2019). Inducible Caspase9-mediated suicide gene for MSC-based cancer gene therapy. Cancer Gene Ther. *26*, 11–16.

56. Wang, C., and Youle, R.J. (2009). The role of mitochondria in apoptosis. Annu. Rev. Genet. *43*, 95–118.

57. Su, H., Lu, R., Ding, R., and Kan, Y.W. (2000). Adeno-associated viral-mediated gene transfer to hepatoma: thymidine kinase/interleukin 2 is more effective in tumor killing in non-ganciclovir (GCV)-treated than in GCV-treated animals. Mol. Ther. *1*, 509–515.

58. Gray, S.J., Foti, S.B., Schwartz, J.W., Bachaboina, L., Taylor-Blake, B., Coleman, J., Ehlers, M.D., Zylka, M.J., McCown, T.J., and Samulski, R.J. (2011). Optimizing promoters for recombinant adeno-associated virus-mediated gene expression in the peripheral and central nervous system using self-complementary vectors. Hum. Gene Ther. *22*, 1143–1153.

59. Montaño-Samaniego, M., Bravo-Estupiñan, D.M., Méndez-Guerrero, O., Alarcón-Hernández, E., and Ibáñez-Hernández, M. (2020). Strategies for Targeting Gene Therapy in Cancer Cells With Tumor-Specific Promoters. Front. Oncol. *10*, 605380.

60. Yun, H.J., Cho, Y.H., Moon, Y., Park, Y.W., Yoon, H.K., Kim, Y.J., Cho, S.H., Lee, Y.I., Kang, B.S., Kim, W.J., et al. (2008). Transcriptional targeting of gene expression in breast cancer by the promoters of protein regulator of cytokinesis 1 and ribonuclease reductase 2. Exp. Mol. Med. *40*, 345–353.

61. Jing, X., Yang, F., Shao, C., Wei, K., Xie, M., Shen, H., and Shu, Y. (2019). Role of hypoxia in cancer therapy by regulating the tumor microenvironment. Mol. Cancer *18*, 157.

62. Yavuz, A.S., Sözer, N.B., and Sezerman, O.U. (2015). Prediction of neddylation sites from protein sequences and sequence-derived properties. BMC Bioinf. *16* (*Suppl 18*), S9.

63. Xiao, X., Li, J., and Samulski, R.J. (1998). Production of high-titer recombinant adeno-associated virus vectors in the absence of helper adenovirus. J. Virol. *72*, 2224–2232.

64. Tomayko, M.M., and Reynolds, C.P. (1989). Determination of subcutaneous tumor size in athymic (nude) mice. Cancer Chemother. Pharmacol. *24*, 148–154.

65. Tsukihara, H., Nakagawa, F., Sakamoto, K., Ishida, K., Tanaka, N., Okabe, H., Uchida, J., Matsuo, K., and Takechi, T. (2015). Efficacy of combination chemotherapy using a novel oral chemotherapeutic agent, TAS-102, together with bevacizumab, cetuximab, or panitumumab on human colorectal cancer xenografts. Oncol. Rep. *33*, 2135–2142.

66. Fischer, A.H., Jacobson, K.A., Rose, J., and Zeller, R. (2008). Hematoxylin and eosin staining of tissue and cell sections. CSH Protoc. *2008*. pdb.prot4986.

67. Choi, H.K., Yessayan, D., Choi, H.J., Schellenberger, E., Bogdanov, A., Josephson, L., Weissleder, R., and Ntziachristos, V. (2005). Quantitative analysis of chemotherapeutic effects in tumors using *in vivo* staining and correlative histology. Cell. Oncol. *27*, 183–190.

68. Porter, A.G., and Jänicke, R.U. (1999). Emerging roles of caspase-3 in apoptosis. Cell Death Differ. *6*, 99–104.

OMTM, Volume *31*

Supplemental information

Inducible caspase 9-mediated suicide gene therapy using AAV6 vectors in a murine model of breast cancer

Subhajit Pathak, Vijayata Singh, Narendra Kumar, and Giridhara R. Jayandharan

SUPPLEMENTAL DATA

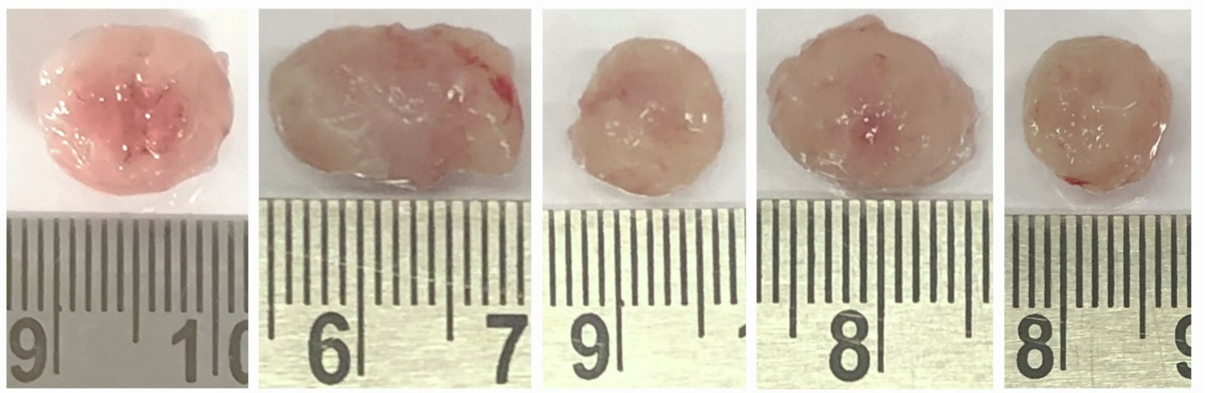

Figure S1. Morphology of breast cancer tumors after suicide gene therapy with AAV6-iCasp9 vectors. Allografted mice with an average tumor volume of ~100-150 mm^3 were administered with AAV6-iCasp9 vectors (5×10^{10} vgs/animal) with or without AP20187 and PBS (mock-treated group). Representative tumor tissues harvested from animals ten days after suicide gene transfer are shown.

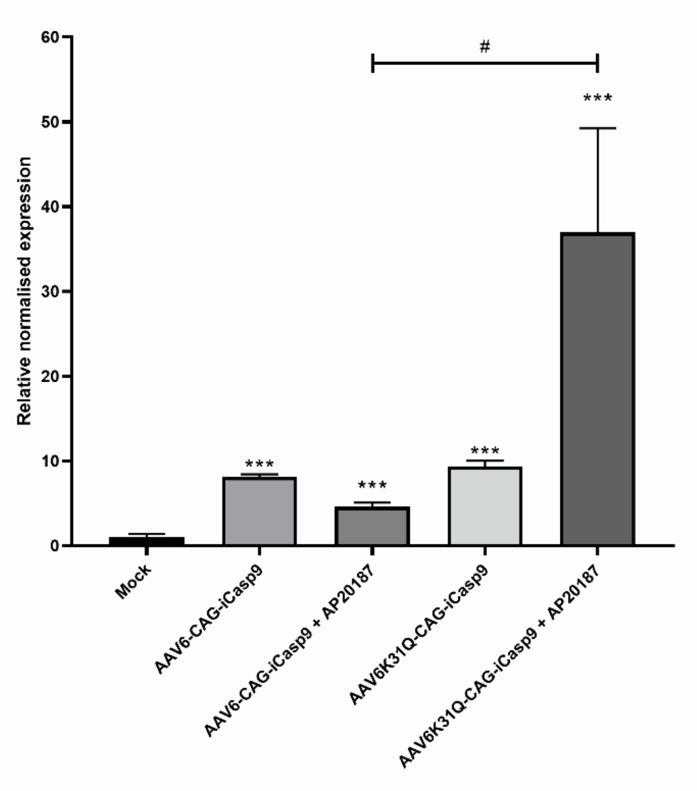

Figure S2. Levels of *iCasp9* after AAV vector administration in breast cancer tissue. An increase in *iCasp9* mRNA levels was observed in breast tumor tissues after administration with AAV6-iCasp9 vectors. Data are from 3-6 technical replicates per group and represented as mean ± SEM. *** $p \leq 0.001$ *vs* mock; $^{\#}$ $p \leq 0.05$ *vs* AAV6-CAG-iCasp9 + AP20187.

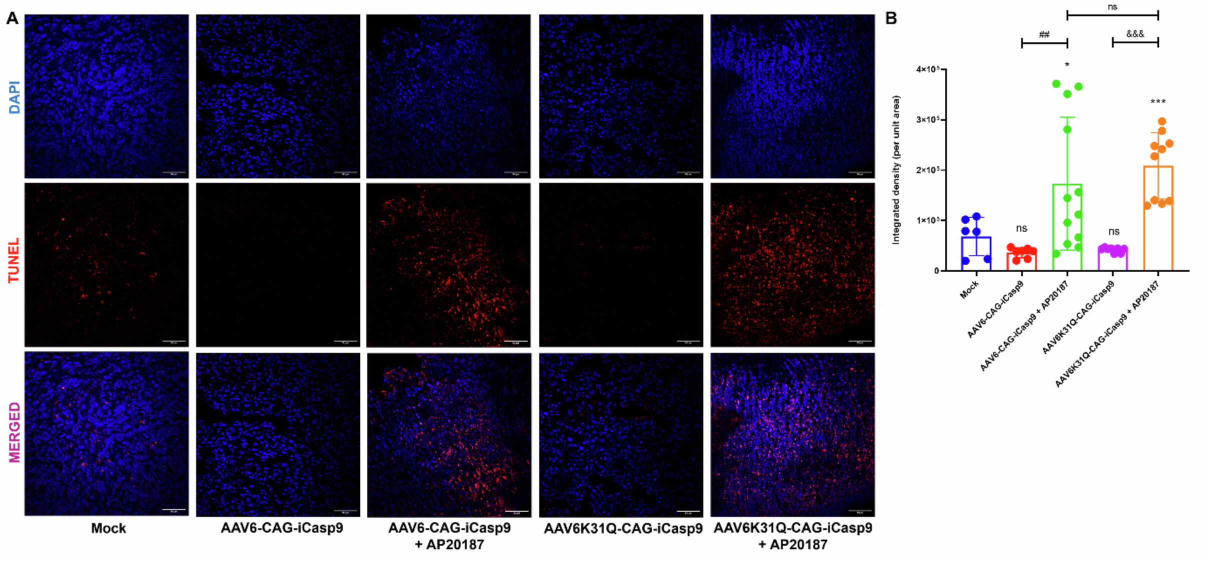

Figure S3. Detection of apoptosis by DNA damage in breast cancer tissue. Ten days after receiving the gene therapy, female athymic$^{nu/nu}$ mice with 4T1 tumors were euthanized. TUNEL assay was performed after surgical removal, fixation, and sectioning of tumors. (A) Micrographs representing the treatment groups (AAV6-CAG-iCasp9 and AAV6K31Q-CAG-iCasp9) with AP20187 showed a clear distribution of apoptotic cells within the tumors when compared to mock-treated animals (magnification 40X, scale bar 50 µm). In AAV6-CAG-iCasp9 and AAV6K31Q-CAG-iCasp9 vector-only administered mice, the absence of AP20187 failed to induce apoptosis. (B) The number of TUNEL-positive cells between various treatment groups was quantified using ImageJ software (* $p \leq 0.05$, *** $p \leq 0.001$, not significant (ns) *vs* mock group; ## $p \leq 0.01$ *vs* AAV6-CAG-iCasp9; &&& $p \leq 0.001$ *vs* AAV6K31Q-CAG-iCasp9). Data are represented as mean ± SD. The data are from 6-12 sections from each treatment group.

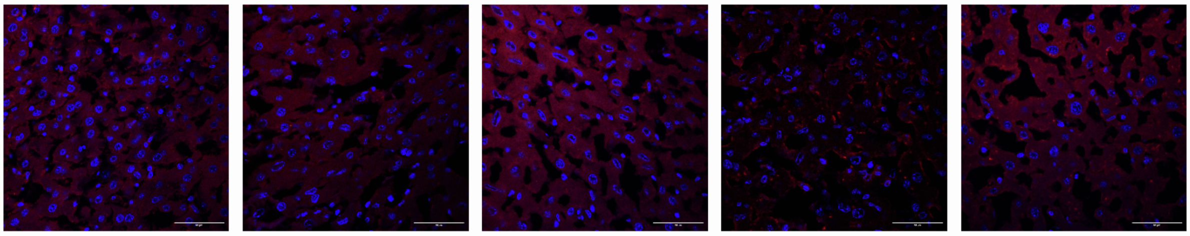

Figure S4. Evaluation of off-target Caspase 9 protein expression in the liver tissue. Liver tissues were harvested from all the treatment groups, and immunohistochemistry was performed as discussed in methods section. The micrographs of the liver sections did not show the activation of Caspase 9 protein in any of the treatment groups (magnification 63X, scale bar 50 μm).
